# Identification of a recessive *PCDH15* nonsense variant in purebred goats with vestibular dysfunction

**DOI:** 10.1016/j.vas.2026.100626

**Published:** 2026-03-18

**Authors:** Eva Petzl, Joana Jacinto, María Climent Aroz, Michael Suntz, Michael Karl, Lutz Plobner, Kaspar Matiasek, Andrea Fischer, Regina Hannemann, Viktoria Balasopoulou, Holm Zerbe, Andreas Brühschwein, Cord Drögemüller, Anna Letko

**Affiliations:** a Clinic for Ruminants with Ambulatory and Herd Health Services, LMU Munich, Oberschleissheim 85764, Germany; b Clinic for Ruminants, Vetsuisse Faculty, University of Bern, Bern 3012, Switzerland; c Institute of Genetics, Vetsuisse Faculty, University of Bern, Bern 3012, Switzerland; d Department of Animal Pathology, Veterinary Faculty, Instituto Agroalimentario de Aragón-IA2, University of Zaragoza, Zaragoza 50013, Spain; e State Institute for Chemical and Veterinary Analysis Freiburg, Freiburg im Breisgau 79123, Germany; f Agrobiogen GmbH, Hilgertshausen 86567, Germany; g Section of Clinical & Comparative Neuropathology, Institute of Veterinary Pathology, Centre for Clinical Veterinary Medicine, LMU Munich, Munich 80539, Germany; h Small Animal Clinic, Centre for Clinical Veterinary Medicine, LMU Munich, Munich, Germany; i Former private veterinary practice for small ruminants and South American camelids, Dr. Regina Hannemann, Tübingen, Germany; j Clinic of Small Animal Surgery and Reproduction, Centre of Veterinary Clinical Medicine, Veterinary Faculty, LMU Munich, Munich, Germany

**Keywords:** *Capra hircus*, Ciliopathy, Neurogenetic disorder, Whole-genome sequencing, Protocadherin

## Abstract

Genetic diseases in goats are less well characterized than in other livestock species. This study reports a previously unrecognized congenital neurological disorder in Bunte Deutsche Edelziege goats and identifies its likely genetic cause. Between 2019 and 2023, a dairy goat farm observed 21 kids with congenital vestibular disease. The affected kids displayed pronounced, sometimes exuberant, wide bilateral excursions of the head and neck, delayed standing, wide-based stance, and poor postural control, while remaining alert and physically strong. Motor coordination improved with age. One retained female displayed mild persistent deficits and suspected hearing impairment in adulthood. Postmortem examinations were unremarkable, and infectious or nutritional causes were excluded. The repeated occurrence and pedigree structure suggested an inherited autosomal recessive disorder. Whole-genome sequencing of two affected kids, compared with large control cohorts, revealed a single rare nonsense variant in exon 5 of the *PCDH15* gene encoding protocadherin-15. This variant truncates approximately 93% of the protein and is homologous to a pathogenic variant that causes Usher syndrome type 1F in humans. Sanger sequencing confirmed homozygosity in affected animals and heterozygosity in obligate carriers. The variant was absent from 90 unrelated goats of the same breed and over 1600 global controls. These findings indicate a naturally occurring Usher syndrome-like disorder in goats, representing the first such report, and highlight the value of genomic diagnostics for disease prevention and comparative research. The study provides a basis for the molecular diagnosis of similarly affected animals, as well as for genetic testing to prevent further cases.

## Highlights

• Nonsense *PCDH15* variant causes vestibular impairment in Bunte Deutsche Edelziege.
• Affected goats show head excursions, wide-based stance, and poor postural control.
• Pedigree information suggests autosomal recessive inheritance.
• First report of naturally occurring Usher-like syndrome in goats.
• Pathogenic variant corresponds to Usher syndrome type 1F-related variant in humans.

## 1 Introduction

Genetic diseases in goats are significantly less studied when compared with other livestock species (Al-Ani et al., 1998; Basrur, 1993). According to the Online Mendelian Inheritance in Animals (OMIA) database (Nicholas et al., 2026), only 13 single-gene disorders have been reported in goats to date, and just 5 of these have at least one likely causal variant identified. These include autosomal recessive disorders such as mucopolysaccharidosis (MPS) IIID (OMIA:000665-9925 and mannosidosis beta (OMIA:000626-9925) in Anglo-Nubian goats, and goitre in Dutch goats (OMIA:000424-9925). Additionally, two dominantly inherited disorders and associated variants have been reported and include myotonia congenita in Tennessee fainting goats (OMIA:000698-9925) and the absence of horns in many goat breeds caused by a complex structural variant, which, in its homozygote state, leads to pseudohermaphrodite females known as polled intersex syndrome (OMIA:000483-9925).

Similarly to genetic disorders, publications and textbooks summarising neurological deficits in goats are scarce (Al-Ani et al., 1998; Basrur, 1993; Smith & Sherman, 2023). Congenital neurological disorders affecting more than one kid on farm are generally associated with environmental causes such as intrauterine infections by teratogenic infectious agents or mineral deficiency. Copper deficiency, for example, is a common problem in dairy goat husbandry and affects all age groups with a great variety of signs (Almeida et al., 2022). Congenital copper deficiency can cause weak kids with varying suckling ability that are often unable to rise, show muscle tremors and pronounced shaking and nodding of the head. Among other things, degeneration and loss of Purkinje cells in the cerebellum are found in histological examination (Almeida et al., 2022; Seaman & Hartley, 1981).

Cases of border disease caused by border disease virus (BDV), on the other hand, are reported rarely in goats. However, they have been described with signs like abortion, poor viability of newborn kids, congenital body tremors, and difficulty in rising and walking (Rosamilia et al., 2014). Natural infections with bovine viral diarrhoea virus (BVDV) are more common but generally result in pregnancy losses and neonatal morbidity and mortality. However, the occurrence of kids with neurologic deficits, such as tremor and ataxia, are reported, as well as the birth of viable persistently infected animals (Bachofen et al., 2013; Passler et al., 2014). Akabane virus in Asia, Africa, the Middle East, and Australia, as well as Cache valley virus in North, Central, and South America and Schmallenberg virus (SBV) in Europe are arthropod-borne orthobunyaviruses that are teratogenic in ruminants, all with the ability to cause brain malformations and arthrogryposis among other signs (Beer et al., 2013; Smith & Sherman, 2023; Uehlinger et al., 2018). Goats have been shown to be less susceptible to several subtypes of bluetongue virus (BTV) and congenital anomalies of the nervous tissue, as described in sheep and cattle seem to be missing, as explicitly reported for BTV-8 (Belbis et al., 2013; Coetzee et al., 2014). Infections with small ruminant lentiviruses (SRLV) can induce leukoencephalomyelitis, causing afebrile, ascending paralysis of legs, involuntary muscle tremors and occasionally torticollis in goat kids. However, clinical signs usually arise between one and four months of age and are not present at birth (Cork et al., 1974; Toplu & Oğuzoğlu, 2023).

The above mentioned inherited neurogenetic lysosomal storage diseases can also cause congenital neurological signs but have so far only been described in Anglo-Nubian goats (Cavanagh et al., 1995; Leipprandt et al., 1996). Goats with MPS IIID might present at birth with inability to rise, a wide-base stance, hyperextension of the limbs, neck tremor, and horizontal nystagmus, as well as ataxia and growth retardation (Jones et al., 1998; Smith & Sherman, 2023). Mannosidosis beta also causes kids to be unable to rise and might display intention tremors and nystagmus; however, they also show contracted tendons with carpal flexion and hindlimb extension as well as varying degrees of facial dysmorphism and bilateral ptosis (Jones et al., 1983; Smith & Sherman, 2023).

The aims of this study were to describe a new clinicopathological phenotype of Bunte Deutsche Edelziege (BDE) goat kids with congenital neurological signs, to investigate the possible genetic cause by a multi-case whole-genome sequencing (WGS) approach, and to evaluate the occurrence of a potential causative allele in the affected population as well as developing a genetic test for diagnostics and disease prevention.

## 2 Material and methods

### 2.1 Herd history and case anamnesis

A newly founded dairy goat farm reported the birth of 21 purebred BDE goat kids with congenital neurological signs between 2019 and 2023. The herd initially consisted of 80 BDE goats purchased from two different farms (A and B) that were both certified unsuspicious of SRLV and *Corynebacterium pseudotuberculosis.* The does and their offspring were served with several bucks without any known genetic relation in the following years. All breeding animals were consistently in good general condition.

The first goat kid with neurological deficits was born in 2019 and examined by the local veterinarian. In 2020, among 108 kiddings, five kids from four female goats (origin: 3 farm A, 1 farm B) exhibited identical neurological signs. In 2021, among 117 kiddings, ten female goats (origin: 6 farm A with buck 1; 3 farm A with buck 2; one goat with unknown origin) gave birth to 12 similarly affected kids. No kiddings occurred in 2022. In 2023, 70 kiddings (female offspring from 2021) resulted in three affected kids. No affected kids were born in 2024 and 2025. One female affected kid, born in 2020, became a pet and was retained for milking.

### 2.2 Clinicopathological examination and samples

All affected kids that were presented to the local veterinarian underwent general and neurological examination. Second opinion neurological examination was performed by video analysis and on site in one kid by a board-certified veterinary neurologist.

Routine brain MRI (1.5 T MAGNETOM Symphony; Siemens Healthineers, Erlangen, Germany) of one alive and one deceased goat kid was carried out and included the following sequences: T2-weighted turbo spin echo (T2W) in dorsal, sagittal, and transverse planes; T2-weighted fluid-attenuated inversion recovery (FLAIR) and T2*-weighted gradient echo in the transverse plane; and T1-weighted spin echo (T1W) and T1-weighted 3D MPRAGE before and after IV bolus injection of the contrast agent gadoteric acid (0.3 mL/kg BW; DotaVision, 0.5 mmol/mL; b.e.imaging GmbH, Baden-Baden, Germany). MRI of the cervical spine of one goat included T2-weighted turbo spin echo (T2W) sequences in sagittal and dorsal planes. MRI images were evaluated in consensus by board-certified specialists in veterinary neurology and diagnostic imaging.

Altogether, across 2020–2023, three affected kids were euthanised for diagnostic reasons and submitted to postmortem examination, along with two spontaneously deceased affected kids. Postmortem examinations of these five kids, all under 2 weeks of age, included gross pathology, tissue sampling, routine histopathology and, in two cases, extended neuropathological examination upon brain trimming according to a modified large animal protocol (Bitschi et al., 2020) adapted for this species. Tissue samples were collected, formalin-fixed, and paraffin embedded. Three-micron thick sections of the brain, spinal cord, dorsal root, ganglia, and nerves as well as spleen, jejunum, liver, lung, heart, and kidney were cut and routinely stained with hematoxylin and eosin. Brain tissue was also stained with luxol fast blue and cresyl echt violet (Klüver-Barrera method). Additional sections of brain stem and cerebellum were immunostained for glial fibrillary acid protein (rabbit anti-GFAP, Z0334, DAKO, Glostrup, Denmark), KCNIP4 (rabbit anti-KCNIP4, ab203831, ABCAM, Cambridge, UK), calbindin (rabbit anti-calbindin-d-28k, C 2724, Sigma, Saint Louis, USA), and ITPR1 (rabbit anti IP3 receptor 1, GTX82887, Genetex Inc., Irvine, USA) using an ImmPRESS® polymer technology (HRP horse anti-rabbit IgG polymer detection kit, Vector Laboratories, Newark, USA) and a diaminobenzidine tetrahydrochloride (DAB, Merck, Darmstadt, Germany) detection system.

PCR for BTV-8 was performed from spleen tissue, PCR for small ruminant pestiviruses (BVD; BVDV) from brain and spleen tissue, and PCR for SBV was performed from brain tissue and accompanied by an ELISA. Furthermore, the goat kids were tested for SRLV infection with a monophase ELISA. In 2020, liver samples from one euthanised kid and from one slaughtered adult female goat were analysed for cobalt, copper, and selenium deficiency. EDTA-blood samples were collected for diagnostic purposes in 2023, including two affected kids, nine related animals (dams and siblings), and three female goats that had never given birth to affected kids.

### 2.3 Genomic analysis

Genomic DNA was obtained from EDTA-blood samples of 14 goats of the herd including two affected animals (adult pet goat; kid born in 2023) using the Promega Maxwell RSC DNA system (Promega, Dübendorf, Switzerland). Subsequently, short-read WGS of the two affected goats was performed using a PCR-free fragment library on an Illumina NovaSeq6000 (Illumina Inc., San Diego, CA, USA). The sequenced reads were mapped and variants were called using the GATK pipeline (Van der Auwera, 2020) with respect to the caprine ARS1 reference assembly (Bickhart et al., 2017) and annotation (GCF_001704415.1). The resulting average read depth of 17 × and 23 × was achieved. Single-nucleotide and small indel variants (SNVs) were called and their functional effects were predicted with SnpEff v5.0c (Cingolani et al., 2012). Variant prioritisation was conducted using publicly available WGS data of 298 unrelated control goats (European Nucleotide Archive accession numbers: PRJEB37122, PRJEB70782, and PRJNA310684).

Based on the described flock structure, a single rare breed-specific allele with an autosomal recessive mode of inheritance was hypothesized as the most probable underlying genetic cause. The SNVs were filtered for private variants present only in the two affected animals and absent from the control genomes. Plink v1.9 (Chang et al., 2015) was used to validate the sex assignment and relatedness of the kids. Furthermore, the allele frequency of detected variants was determined in a global cohort of 1372 unrelated caprine genomes available from the VarGoats project (Denoyelle et al., 2021). The impact of the identified variants on the encoded proteins was predicted using the in silico prediction tool MutPred2 (Pejaver et al., 2020). For comparative analysis across species using protein sequence alignments, the following NCBI protein accessions were used: XP_005698207.2 (*Capra hircus*), XP_059737953.1 (*Bos taurus*), NP_001371069.1 (*Homo sapiens*), XP_038293589.1 (*Canis lupus familiaris*), NP_001136214.1 (*Mus musculus*), XP_020929194.1 (*Sus scrofa*). The protein sequence was visualized using the Protter web application (Omasits et al., 2013).

Finally, the candidate causal variant in the *protocadherin-15* (*PCDH15)* gene was validated and genotyped in all 14 BDE goat samples by Sanger sequencing of PCR amplicons using the following primers: forward: 5′-GGCATCCCAGCAAATCTCAGC-3′ and reverse: 5′-GCTTTCATGCTTGAATGTGGGT-3′. To investigate the candidate variant allele occurrence in unrelated breed controls, a total of 90 BDE goats, originating from six different herds in Germany, were genotyped by the veterinary diagnostic laboratory Agrobiogen, Germany, using a KASP assay.

## 3 Results

### 3.1 Clinicopathological assessment reveals a novel vestibular disorder in goat kids

**Fig. 1:**
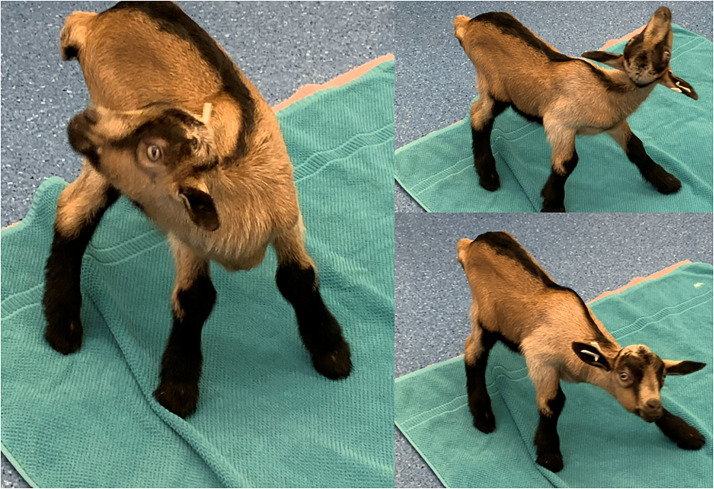
Affected goat kid displaying characteristic signs of bilateral vestibular disease: a wide-based stance and wide bilateral head and neck excursions.

All affected goat kids showed congenital neurological deficits. The most prominent clinical feature displayed by all kids were wide bilateral head and neck excursions that seemed vigorous and exuberant at times and were present from birth (Supplementary Video S1; Fig. 1). Although the animals appeared alert, well-oriented, and physically strong, they were unable to maintain adequate postural control and head coordination required to suckle from the doe. When bottle-fed, all affected goat kids demonstrated an intact suckling reflex and good appetite. However, the excitement from feeding sessions markedly intensified head excursions and overshooting movements, necessitating manual stabilization of the head and guided placement of the bottle. In addition to the wide stance, affected kids exhibited further signs of balance deficits. They were prone to fall when lightly pushed and had difficulty righting themselves from lateral to sternal recumbency. They displayed difficulty in jumping, climbing, and maintaining a straight-line gait (Supplementary Video S1). The neurologic signs were characteristic of bilateral vestibular disease (Fig. 1).

Complete neurological examination of one kid showed normal mental status, no signs of vision impairment, a very broad, wide-based stance in all four limbs, truncal sway, and wide head and neck excursions to either side, consistent with a bilateral vestibular deficit (Supplementary Video S2). Other findings were mildly delayed postural reactions and increased extensor muscle tone in the thoracic limbs. Additionally, the kid demonstrated frequent, subtle muscle fasciculation involving various muscle groups. Cranial nerve examination was unremarkable. No signs of nystagmus or strabismus were noted in any position (standing, lateral or dorsal recumbency).

MRI scan of the brain parenchyma, middle and inner ear regions and the spinal cord in two affected kids failed to identify any structural lesions. Liver analysis revealed physiological levels of the trace elements, and all samples were negative for the tested pathogens. Antibodies against SBV were detected in one animal and all goats were seronegative for SRLV infection.

All of the 21 affected goats showed good general condition, normal growth and were, with the exception of 8 animals (3 euthanised for diagnostic reasons, 4 spontaneously deceased, 1 retained for milking), raised until they reached slaughter weight. Over time, motor coordination improved, and the kids were capable of independent bucket feeding. The four affected kids that died in the first weeks of life, showed signs of acute clostridiosis and pneumonia that was believed to be associated with the side effects of bottle and bucket feeding under farm conditions. One female kid became a pet and was retained for milking. This doe exhibited normal physical development, above-average milk production, and delivered an unaffected kid. Over time, its abnormal head movements became subtle, and its gait appeared straight and stable. Nevertheless, she demonstrated persistent difficulty passing by a 60 cm step into the milking parlour. In that daily situation, the animal showed marked distress, targeting the step from all sides but never attempted to jump until manual assistance was given by the farmer. Furthermore, the doe appeared unresponsive to auditory stimuli and exhibited sporadic, abnormally loud and strange vocalizations. No signs of spatial disorientation within the stable were reported.

Postmortem examination of five kids revealed a good body condition and physiological growth. No morphological abnormalities were found in any of the examined kids over the years, neither in the nervous system nor in the rest of the body. No changes associated with middle ear cavities, vestibulocochlear nerves and respective nuclei, cerebellar peduncles, and vestibulospinal tracts, longitudinal medial fasciculus, cerebellar nuclei, foliary and deep cerebellar white matter, and cerebellar cortex of all functional compartments (vestibulocerebellum, spinocerebellum, and cerebro-ponto-cerebellum) were apparent in the two kids subjected to extended neuropathological examination. All cell markers were within normal limits and there were no signs of neuronal, glial or vascular changes.

Taken together, the clinicopathological findings in the examined BDE goats were consistent with a congenital bilateral vestibular disease, showing similarities to Usher syndrome in humans.

### 3.2 Genomic analysis identifies a novel candidate variant

DNA from EDTA-blood samples of two affected animals, as well as 9 samples of related animals (does and siblings), and 3 samples of does that had never given birth to affected kids were analysed. WGS of the two cases was carried out and stepwise variant filtering was performed for both heterozygous and homozygous variants (Table 1). However, autosomal recessive mode of inheritance was deemed more likely, given that all parents and other kids in the flock were apparently unaffected. Furthermore, the proportional identity-by-descent value was found to be 0.32 indicative for half sibs, thereby confirming close relatedness within the flock.

**Table 1:** Results of variant filtering using the whole-genome sequence data from both affected kids and 298 controls of various other breeds as well as the VarGoats global cohort (*n* = 1372).

Filtering step	Homozygous variants	Heterozygous variants
All shared autosomal variants in the two affected kids	2,656,821	2,771,647
Private shared variants in the two affected kids after comparison with the 298 public caprine controls	142	16,023
Private protein-changing variants in the two affected kids after comparison with the 298 public caprine controls	1	92
Private protein-changing variants after comparison with the VarGoats global cohort	1	40
Private protein-changing variants after comparison with the VarGoats global cohort in a functional candidate gene	1	1

Inspection of shared SNVs and indels revealed only one private heterozygous missense variant in a functional candidate gene *ADGRV1* encoding adhesion G protein-coupled receptor V subfamily member one*.* This substitution (XM_018050044.1:c.12212G>*A*; XP_017905533.1:p.(Arg4071Gln)) was predicted benign by the in silico prediction tool MutPred2 (score 0,19).

**Fig. 2:**
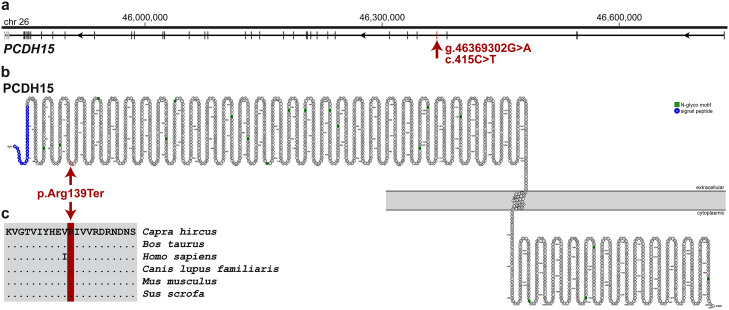
Genetic analysis of the nonsense variant in goats affected by Usher-like phenotype. (a) *PCDH15* gene structure showing the location of the candidate variant XM_005698150.2:c.415C>*T* on chromosome 26, exon 5 (red arrow). (b) Schematic representation of the caprine PCDH15 transmembrane protein showing the position of the XP_005698207.2:p.(Arg139Ter) stop-gain variant (red circle and arrow) that is predicted to truncate ∼93% of the wildtype protein. (c) Multiple sequence alignment of the PCDH15 protein encompassing the region of the affected residue reveals complete evolutionary conservation across species.

Considering the recessive mode of inheritance, both animals were homozygous for the alternative allele at 142 autosomal loci, while all 298 control goats of various other breeds were homozygous for the reference allele at these loci. More than half of these loci (79/142) colocalize on chromosome 26 within 2.7 Mb region of shared homozygosity. Only one of these variants was predicted to be protein-changing with high impact on the encoded protein and was also absent from the global cohort of 1372 goats (Table 1). This homozygous nonsense variant was found on chromosome 26 (NC_030833.1:g.46369302G>*A*) affecting exon 5 of the *PCDH15* gene (XM_005698150.2:c.415C>*T*), which is common to all isoforms (Fig. 2a). It results in a premature stop codon truncating ∼93% of the wildtype PCDH15 protein (Fig. 2b) including functionally important domains (XP_005698207.2:p.(Arg139Ter)). MutPred2 prediction showed moderate evidence for loss of function probability (score 0.37) and highlighted likely disrupted molecular features: catalytic site (*p* = 0), protein–protein interaction interface (*p* = 0), iron binding site (*p* = 0), sulfation site (*p* = 0), and proteolytic cleavage (*p* = 9.99 × 10^–06^). Furthermore, comparative analysis showed that the affected amino acid is located in a region highly conserved across species (Fig. 2c) and a homologous pathogenic variant (NM_001384140.1:c.400C>*T*; NP_001371069.1:p.Arg134Ter) has been described in several human patients (ClinVar VCV000218194.12).

Sanger sequencing was used to confirm the variant allele and to genotype close relatives of the affected animals and further unrelated control goats. Notably, only the two affected BDE goats showed homozygous alternative genotype (alt/alt) and six unaffected dams known to have produced affected offspring were heterozygous (ref/alt) for the alternative allele (Table 2).

**Table 2:** Association of the nonsense variant in *PCDH15* with the vestibular dysfunction phenotype in Bunte Deutsche Edelziege goats.

	*PCDH15*: c.415C>*T* genotype		
	ref/ref	ref/alt	alt/alt
Affected goats			2
Obligate carriers^a^		6	
Unaffected herdmates^b^	4	2	
Other unrelated BDE goats^c^	90		
Sequenced control goats from various breeds^d^	1670		
Total	1764	8	2

a Parents of affected animals were classified as obligate carriers.

b Unaffected goats including siblings from the same farm.

c Same breed controls with unknown phenotypes.

d Global cohort of publicly available unrelated caprine whole-genome sequences.

## 4 Discussion

In our study, a comprehensive genetic evaluation of two purebred BDE kids with congenital vestibular dysfunction was performed. The occurrence of a low number of affected animals across several years in different kidding seasons suggested a possible genetic cause, most likely autosomal recessive. The clinicopathological findings were consistent with a congenital bilateral vestibular disease and indicated a disorder similar to Usher syndrome (USH) in humans (de Lahunta & Glass, 2009; D'Esposito et al., 2025).

USH is considered a ciliopathy (D'Esposito et al., 2025; Fuster-García et al., 2021), causing the majority of combined deaf blindness in humans younger than 65. The autosomal recessive inherited disease is clinically and genetically heterogeneous and is characterised by sensorineural hearing loss (SNHL) and retinitis pigmentosa (RP) with or without vestibular dysfunction (Velde et al., 2022). USH is classified in four clinically defined subtypes with 16 associated gene variants that are known today (D'Esposito et al., 2025; Fuster-García et al., 2021). The associated genes encode key structural proteins for the development and function of auditory and vestibular sensory cells in the inner ear and photoreceptors of the retina. Therefore, the associated gene variants cause destruction and disruption of different structural proteins in ciliary cell function, resulting in different entities and onset of clinical signs (Castiglione & Möller, 2022).

WGS analyses of the affected BDE goats identified a rare nonsense candidate causal variant in the *PCDH15* gene. This variant was homozygous in the genomes of both affected kids and heterozygous in six females with similar cases among their offspring, consistent with Mendelian monogenic recessive inheritance. Heterozygous carriers were neither found in the control group of 1670 sequenced goats of various breeds, nor in the control group of 90 BDE goats. The alternative allele appears to be absent from the general population, which, together with the observed Mb-sized flanking region of autozygosity on chromosome 26, suggests that the mutation occurred more recently and that local inbreeding practices may have contributed to the emergence of affected animals. However, the farmer reported that affected goat kids were born to animals from two origin farms and that bucks were exchanged every breeding season with newly acquired purebred animals. This might indicate a possible pool of carriers in the BDE breed. Unfortunately, breeding records were unavailable for detailed pedigree analysis so that this hypothesis remains unconfirmed.

It was not possible to perform experimental RNA analysis, as the affected animals were unavailable for further sampling at the time of genomic analysis, and thus consequences of the *PCDH15* nonsense variant could not be accurately validated at the transcript level. Nevertheless, according to the Genome aggregation database (Karczewski et al., 2020), 66 variants in the *PCDH15* gene have been classified as pathogenic or likely pathogenic in human patients. A homologous pathogenic variant of the herein described caprine variant has been reported in human patients with USH type 1F (OMIM:602083; ClinVar VCV000218194.12). Additionally, a single shared, private, heterozygous missense variant in the *ADGRV1* gene was found in both analysed affected animals. Homozygous variants in the human *ADGRV1* gene are associated with USH type 2C (OMIM:602851) and have been associated with febrile and afebrile seizures (Guan et al., 2023). The caprine variant was also predicted to have a benign effect on the encoded protein. Therefore, we assumed that this variant is unlikely to be disease-causing.

USH type 1F in humans is caused by pathogenic variants in the *PCDH15* gene and is generally characterised by congenital profound SNHL, signs of bilateral vestibular areflexia, and early onset of RP. However, rare exceptions with residual vestibular and auditory function have been described (Wafa et al., 2021). The extracellular adhesion protein PCDH15 belongs to a family of integral membrane proteins that mediate calcium-dependent cell-cell adhesion, playing an essential role in normal retina, cochlea, and vestibular function (Amorim et al., 2024; Castiglione & Möller, 2022). Pathogenic variants in the *PCDH15* gene cause defects of planar polarity of hair cells in the cochlea and in type 1 and type 2 vestibular hair cells (Amorim et al., 2024; Kaushik et al., 2025), disabling mechanotransduction and therefore causing loss of hearing and balance (Ivanchenko et al., 2024). However, the mechanisms of retina degeneration caused by *PCDH15 v*ariants have not been fully understood. It is assumed that PCDH15 is a component of the calyceal processes that surround the bases of photoreceptor outer segments and maintain their integrity (Ivanchenko et al., 2023).

Many mouse models for USH type 1 were identified by signs of vestibular dysfunction resulting in characteristic circling and head-tossing behaviour of the animals and descriptive names such as shaker1 (sh1), deaf circler (dfcr), waltzer (v), Ames waltzer (av), and Jackson shaker (js) (Williams, 2008). These characteristics are highly similar to the clinical signs noted in the herein described goats. Furthermore, the delayed acquisition of independent standing (Lickliter, 1985) and the prominent difficulties in postural control in the neonatal goat kids, as well as the marked improvement over time shows common characteristics to human USH type 1 patients. In infants, bilateral vestibular areflexia becomes evident in delayed gross motor development generally associated with neonatal hypotonia and delays in sitting, crawling, and walking. During childhood, balance deficits are more subtle, becoming apparent in challenging situations (Castiglione and Möller (2022). Improvement of gross motor development during the juvenile phase has also been described in an USH type 1 pig model (Grotz et al., 2022) and is coherent with growing visual and somatosensory compensation for missing vestibular clues (Thomas, 2000; Wafa et al., 2021). Furthermore, the adult pigs (Grotz et al., 2022) showed no obvious gait instability but had difficulties to pass obstacles that required vertical movement and therefore additional balance, similarly to the herein described affected adult goat. Naturally occurring variants in livestock like the one described here may provide complementary insights for translational research, as shown recently through gene therapy in a calf with classic maple syrup urine disease (Wang et al., 2025). Therefore, preserving samples before elimination of the alternative allele from the goat population could support evaluation of this caprine model for human USH syndrome and emerging therapies.

Unfortunately, the *PCDH15* variant was detected long after the clinical assessment and postmortem examinations of the goat kids and no affected kids were born in 2024 and 2025, probably because chosen breeding bucks did not carry the identified variant. Due to these reasons neither advanced functional (e.g., auditory brainstem response, electro-retinography) nor structural assessment (e.g., histopathology as scanning electron microscopy) of the auditory and visual system was possible. However, the clinical picture of a bilateral congenital vestibular disease, in absence of any macroscopic and microscopic changes in the vestibulocochlear nerves, brain stem, and cerebellum, and the autosomal monogenic recessive inheritance with the identified candidate variant in the *PCDH15* gene, strongly suggest a disease pattern with parallels to USH type 1F seen in humans. Although it could not be confirmed, the unusual vocalisation and the indifference of the affected adult pet goat to auditory stimuli could be interpreted as indicative of impaired hearing ability. Interestingly, no signs of spatial disorientation due to possible visual impairment of the goat within the stable were reported. Considering the fact that USH type 1F patients may retain functional vision into their 30 s or even 40 s (D'Esposito et al., 2025) with marked correlation of visual field impairment with declining postural stability (Wafa et al., 2021), it is possible that this relatively young, clinically inconspicuous animal was not yet suffering from vision impairment or that it was well compensated in the familiar stable environment.

In conclusion, we identified a most likely pathogenic variant in the *PCDH15* gene in goats affected by a recessively inherited congenital vestibular and suspected auditory impairment resembling the human USH type 1F. The findings inform BDE goat breeders and veterinarians about the possible cause of a congenital neurogenetic disorder. DNA testing enables precise molecular diagnosis of animals displaying similar signs and prevention of further affected animals by exclusion of risk matings. We demonstrate the value of precision diagnostics in elucidating rare diseases and monitoring breeding populations for deleterious alleles. Furthermore, this study emphasises the practicality and growing importance of targeted genomic analyses in veterinary medicine and comparative biomedical research through naturally occurring animal models.

## Ethics approval

All animals in this study were examined with the consent of their owners and handled in accordance with good ethical practice. All live animals were blood sampled for diagnostic purposes on the farm to determine the cause of the disease. All other samples were obtained at postmortem examination of affected animals after euthanasia on humane grounds and were submitted by the owner for laboratory diagnostic investigation.

## Data and model availability statement

All whole-genome sequences associated with this study are deposited at the European Nucleotide Archive (https://www.ebi.ac.uk/ena/) under study accession numbers PRJEB70782, PRJNA310684, and PRJEB37122. Sample accession numbers of the two affected goats are ERS21362004 and ERS21362005.

## Funding

The authors declare that no funds, grants, or other support were received during the preparation of this manuscript.

## CRediT authorship contribution statement

**Eva Petzl:** Writing – review & editing, Writing – original draft, Visualization, Project administration, Investigation, Conceptualization. **Joana Jacinto:** Writing – review & editing, Writing – original draft, Investigation. **María Climent Aroz:** Writing – review & editing, Validation, Investigation. **Michael Suntz:** Writing – review & editing, Investigation. **Michael Karl:** Validation, Resources. **Lutz Plobner:** Validation, Resources. **Kaspar Matiasek:** Writing – review & editing, Investigation. **Andrea Fischer:** Writing – review & editing, Visualization, Investigation. **Regina Hannemann:** Writing – review & editing, Supervision, Resources. **Viktoria Balasopoulou:** Writing – review & editing. **Holm Zerbe:** Writing – review & editing, Supervision, Resources. **Andreas Brühschwein:** Writing – review & editing, Investigation. **Cord Drögemüller:** Writing – review & editing, Supervision, Project administration, Funding acquisition. **Anna Letko:** Writing – review & editing, Writing – original draft, Visualization, Software, Project administration, Methodology, Investigation, Formal analysis, Data curation, Conceptualization.

## Declaration of competing interest

The authors declare the following financial interests/personal relationships which may be considered as potential competing interests:

Lutz Plobner reports a relationship with Agrobiogen GmbH that includes: employment. Michael Karl reports a relationship with Agrobiogen GmbH that includes: employment. If there are other authors, they declare that they have no known competing financial interests or personal relationships that could have appeared to influence the work reported in this paper.

## Appendix Supplementary materials

**Supplementary Video 1** Collection of video sequences showing the affected goat kids in a barn environment. Image, video 1 dummy alt text

**Supplementary Video 2** Affected goat kid displaying wide-based stance, truncal sway, and wide head and neck excursions to either side, consistent with a bilateral vestibular deficit. Image, video 2 dummy alt text
